# Print, Play, and Learn: Cataloging Card and Board Games for Medical Education From 1980 to 2025

**DOI:** 10.7759/cureus.99203

**Published:** 2025-12-14

**Authors:** Michael Cosimini, Aryana Zarandi, Sarah L Edwards, Mikaela L Stiver, Vincent Chan, Odolphe Augustin, Bruce Blain, Teresa M Chan

**Affiliations:** 1 General Pediatrics, Oregon Health & Science University, Portland, USA; 2 Health Sciences, McMaster University, Hamilton, CAN; 3 Emergency Medicine, University Hospital of Nottingham NHS Trust, Nottingham, GBR; 4 Cellular and Physiological Sciences, University of British Columbia, Vancouver, CAN; 5 Pulmonary and Critical Care, Thomas Jefferson University Hospital, Philadelphia, USA; 6 Internal Medicine, Johns Hopkins Bayview Medical Center, Baltimore, USA; 7 Law and Business, Toronto Metropolitan University, Toronto, CAN; 8 Emergency Medicine, Toronto Metropolitan University, Toronto, CAN

**Keywords:** analog games, continuing medical education, gamification, graduate medical education, medical education, serious games

## Abstract

Background

Card and board games are increasingly described in the medical education literature, but games in the literature are not always available for use by educators. Since the last survey of games for medical education, new funding and distribution technologies have reduced barriers to sharing games, and we hypothesize that more games are available than described in the literature and that new technologies have contributed to this availability. We aim to describe the current landscape of games beyond the literature to facilitate their use and study.

Methods

For this study, we curated a list of sites where games are available for download and/or purchase by searching for sites associated with the games from an earlier review. We searched these sites over a three-year period to build a catalog of games published in or before 2024 that were designed for physicians and/or physician-track learners. We described the audiences, content areas, technologies used for distribution, and other details about the games.

Results

We identified 224 games, of which 87 met the inclusion criteria. The number of games increased year-over-year from 2013-2019, and the peak year was 2023. Popular game topics included infectious disease (n=15), anatomy (n=11), neurology (n=10), pediatrics (n=10), and emergency medicine (n=9). Games were often shared using printable files (34) and print-on-demand services (23). Only 31 (36%) games had an associated academic publication.

Conclusions

The number of medical education games has substantially increased in the last decade, facilitated by the adoption of print-on-demand and the sharing of printable files. Gaps in content area and target audience still remain, and we encourage educators to employ funding and distribution technologies to facilitate sharing games more widely.

## Introduction

Innovative medical educators are always looking for new ways to engage their trainees, and educational games are an increasingly popular tool for facilitating active learning. Students and educators interested in card and board games for medical education find a lack of available games a barrier to use for both teaching and learning [1], and games studied for medical education are often not available to the readers [2]. The last survey of the available games for medical education in 2007 found 17 games for undergraduates and one publication describing a game for postgraduate learners [3]. Surveying the current landscape of games is important to help match educators with games and to identify content area gaps where more could be developed. 

Outside of medicine, games are widely adopted in education, with evidence in a variety of fields. Among grade-school teachers in the US, 82% teach with non-digital games [4]. Card and board games are also commonly studied in higher education [5], and evidence for the efficacy of card and board games exists in many areas [6]. Notable are studies where games have compared positively to active controls in impacting knowledge [7-9] and attitudes [8,9] in randomized studies. 

Since the landscape was last surveyed, technological innovation has reduced barriers to the funding and distribution of games, including print-on-demand services, which will manufacture a professional-quality copy of a game to order, and crowdfunding services, which are used to fund, promote, and act as a pre-purchase platform for games [10]. Print-on-demand services and crowdfunding both allow educators without sufficient resources to fully fund the manufacturing of games to share their creations in a more professional presentation. In particular, crowdfunding is a common method [11] to fund the development of games, and we anticipated it would be adopted to fund games for medical education. Longer standing is the sharing of printable files, sometimes called “print and play,” where files are often freely distributed for users to assemble their own copy of a game at home. 

We limit this study to card and board games in line with the inclusion criteria of the literature review used to generate the sources of games used for this study [2]. Unlike other medical education games or gamified educational experiences using digital platforms, augmented reality, or virtual reality, analog games do not require additional hardware and do not lose functionality as systems change. This makes them well-suited to ongoing indexing as educational tools.

We hypothesize that many games are not described in the peer-reviewed literature, and that educators will have adopted new technologies to share games. We aim to describe the landscape of card and board games for medical education and hope this data will (1) help educators find games for their own teaching needs, (2) identify gaps in the landscape, and (3) inspire educators to share the games they have made with the larger community.

## Materials and methods

We conducted a study identifying card and board games available for medical education from a broad range of sources. We performed this study in four phases: (1) using the published literature to identify online repositories that distribute, list, or sell games, (2) searching inside the repositories for games, (3) reviewing games for inclusion, and (4) extraction and analysis of data from these sources.

Step 1: Identifying repositories listing or selling games for medical education

We created a list of sources that list or sell games by searching for games described in the medical education literature. After a calibration exercise, we reviewed full-text articles from Edwards et al. and recorded any description of where games were available [2]. Additionally, where a game title was provided, we searched online with Google for availability. This was done by searching for the game's name alone and the name in quotation marks, plus the search term “game.” We reviewed the first 30 listings and recorded relevant game-specific websites, publishers' webpages, print-on-demand sites, crowdfunding platforms, and designers' web pages. After this process, we continued to add sources over time by sharing lists of games in online communities dedicated to board games or medical education and requesting additional titles or sources for games.

Step 2: Searching inside the identified repositories for further games 

We searched identified sites for games in March 2023, January 2024, and February 2025. Search strategies were site-specific. Generally, we searched websites for “education,” “educational,” and “medical,” and for educational game publishers, we reviewed their full catalogs. We recorded titles and descriptions of games thought to have the potential to meet inclusion criteria. 

Step 3: Reviewing for inclusion in the study

After deduplication, two investigators reviewed available information online about the games against study inclusion criteria (AZ, VC, MC, BB), and ties were broken by a third investigator (MS, SE). We included card and board games designed for the purpose of the education of physician track learners, including medical students, residents, fellows, or attending physicians, which were available for use beyond the original designers. We included games that appeared to have been available at any point in time, not just games available during the study. Availability could be either a physical product or a digital file to print. Expansions to an existing game were not included if they required a base game for use. We did not strictly define what constitutes a game, generally accepting the designation by designers. However, when uncertain, we referenced Jane McGonigal's first three criteria for defining a game: (1) having goals, (2) rules, and (3) a feedback system [12].

Step 4: Data extraction and analysis

To describe the spectrum of games being developed, we recorded details about the games, including player count, play time, content area, and audience. We generated a timeline of games released by year.

## Results

Identifying games and reviews for inclusion

**Figure 1:**
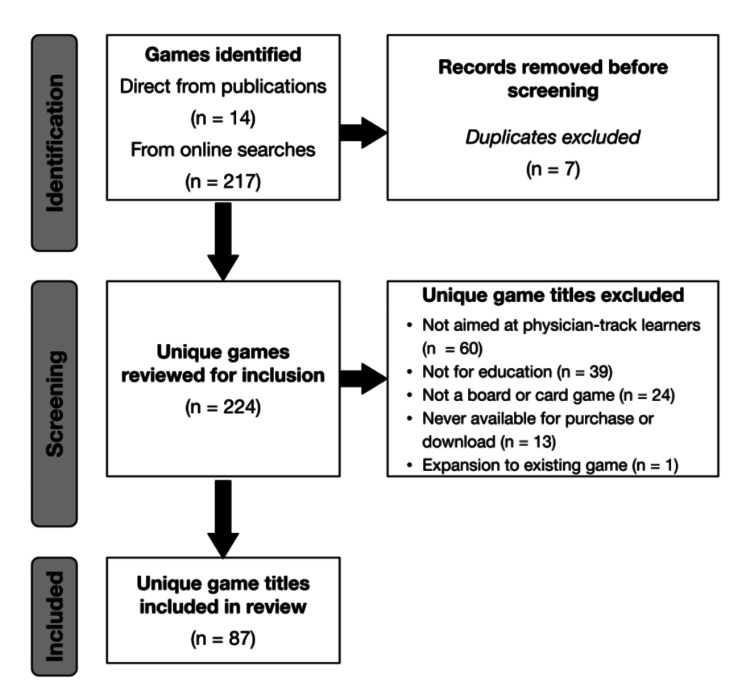
Flow diagram of game review for inclusion.

We searched 27 sites and identified 217 games. Seven games were identified directly from the original publications only. Of the 224 total unique games, 87 games met the inclusion criteria. A flow diagram of the review process is presented in Figure 1.

Timeline of game release and technologies used

**Figure 2:**
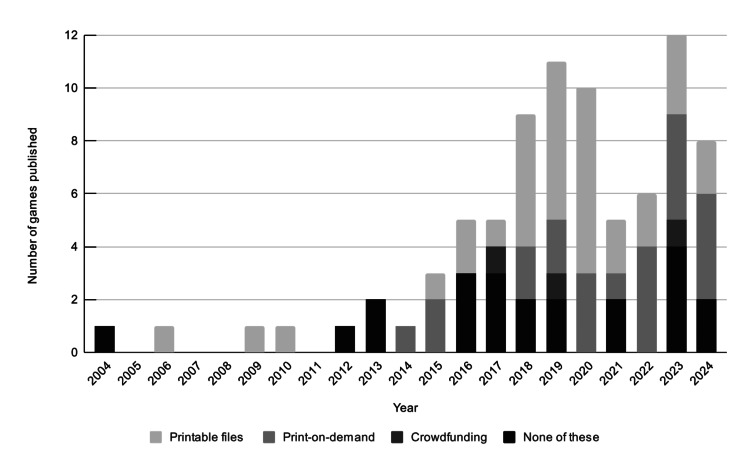
Bar chart showing the number of games published from 2004 through 2024. Three additional games published in 1980, 1992, and 1995, respectively are not shown. Technology used to fund or distribute the games are delineated by shade.

The year of publication was available for 85 games, the first of which was published in 1980. The number of games published peaked at 12 games in 2023 after a drop in 2021 and 2022, likely related to the COVID-19 pandemic and challenges around developing and implementing in-person teaching (Figure 2). A timeline of games published is presented in Figure 2. Games were shared as printable files in 34 cases, often as supplements in places such as MedEdPORTAL (n=14). Print-on-demand services were used for 23 games, including The Game Crafter (n=19) and Drivethrucards (n=4). Only three games used crowdfunding. Another source of the increase in games is the UK-based publisher Focus Games, which contributed 21 games. Of 87 games, 31 (36%) had at least one associated publication [13-40].

Description of included games

A variety of content areas were covered in games; the most common clinical areas were infectious diseases (n=15), neurology (n=10), pediatrics (n=10), and emergency medicine (n=9). Anatomy (n=11) was another common area and often aimed at medical student learners. Games were used to teach communication skills (n=6), task prioritization (n=4), or systems-based practice (n=8), including prevention of errors, infection control, and hospital patient flow.

Many games did not explicitly specify the learners for whom they were designed. Among those that did, medical students were the most common audience at 50 (57%), residents or fellows had 31 (36%), and attending physicians also had 31 (36%). The number of players a game could support ranged widely, from as low as one player in 14 games to as high as 200 for a large simulation, Friday Night at the ER, which uses multiple copies of the game. The most supported player counts were two (62%), three (77%), four (84%), and five (62%) players. Appendix 1 contains a full list of games, content areas, audience, player counts, and playtimes.

## Discussion

The number of card and board games available for medical education has substantially increased, facilitated by distribution via printable files and print-on-demand services. Crowdfunding has not played a substantial role. The 87 games found in this review demonstrate the significant growth in the field compared to the last systematic survey of card and board games in 2007 [3]. In 2007, few games were available for play in any format. Only two of the 14 publications cited in this review included an adequate description to fully recreate the games [41,42], and none provided printable files. Publishing specific details about educational games is imperative for maintaining rigor and advancing the field [43].

While games described in the literature are often not available for use, we found here that the reverse is also true, that games available for use were uncommonly described in the literature, with 36% having an associated publication. This could be due to barriers to study, such as a lack of time, money, methodological expertise, and mentoring [44], or the study may not be a priority for the designers. We believe all games described in the literature should be made available in some form for scientific rigor. In contrast, not all games made available for use need to have a backing in the literature. 

Uptake of new technologies has been critical for the distribution of new games, including sharing printable digital files, and ease of access to print-on-demand services. We identified 34 games that were disseminated using printable files, a method that enables low-cost distribution for both designers and end users, and eliminates most barriers to international distribution [45]. However, there are significant limitations in the types of games that can be reasonably shared as digital files. Considerations include how much material needs to be printed and assembled, access to printing services and the quality thereof, and an inability to include non-printable game components such as dice or pawns [46]. We found 23 unique games for which print-on-demand services were used to facilitate distribution. Print-on-demand services enable high-quality game printing and assembly, but they carry a higher cost to the end-user than mainstream games produced by companies with large-volume operations [46]. Crowdfunding, a widely acknowledged tool for supporting innovation [44], was only used for three games. Crowdfunding technology is a popular tool in the hobby game industry for raising the necessary funds to cover high-volume print runs that reduce the unit cost; however, the complexity of crowdfunding processes and the relatively modest size of the potential market for niche medical education games may be among the barriers leading to its infrequent use. Additionally, successful crowdfunding requires marketing skills that might not be present in medical educators.

Almost half of the card and board games in this review covered content related to infectious diseases, pediatrics, neurology, or emergency medicine, meaning that there are numerous opportunities to develop new games focusing on less commonly represented topics or topics not seen at all, such as surgery. The reasons for the concentration of games likely have multiple factors. Individuals and publishers may contribute, as four of 10 neurology games and four of 15 infectious disease games are each from a single designer. Similarly, one publisher published six of the eight systems-based practice games. Games may also be focused on areas traditionally perceived as difficult for undergraduate medical education, such as anatomy and neurology [47,48]. Some topics might also be particularly well-suited to tabletop game mechanics, such as anatomy, where these mechanics are used to explore the relative position of three-dimensional structures [49]. 

Games were largely designed to be used with medical students and/or residents, with fewer options intended for continuing medical education. Even so, games designed for continuing medical education represented a higher proportion in this study (36%; n=31) compared to our previous review that only included games published in traditional, peer-reviewed journals (19%; n=14) [2]. 

Some limitations to consider are that we limited our pool of games to those that are designed for medical students, residents, or post-residency medical trainees, and we are not describing a larger pool of games designed exclusively for other health professions, including the large body of games for nursing education. We worked from the English language literature and performed searches in English, limiting the pool of games discovered. We are also limited by available information online and in the literature describing games, as we selected and described the games. Another limitation of our review is the reliance on recent literature to identify our pool of sites. Omissions of some older games could have led to an inflated sense of recent growth. While we repeated our search several times over three years and used diverse methods to find sources, there is always the potential to miss games. Nevertheless, we believe that this novel approach was appropriate for our aim of describing the contemporary landscape of card and board games designed for medical education, including games that are not found in traditional academic literature.

## Conclusions

Many card and board games for medical education have been developed and made available beyond what is described in the literature, in part through the adoption of new distribution technologies. Continued study of efficacy remains important as games are developed and used, given the limitations of the current literature. We encourage educators to continue designing and studying these games and to use newer distribution technologies in conjunction with publishing in traditional peer-reviewed journals to facilitate awareness and accessibility for use in other contexts worldwide.

## Appendices

Appendix 1 

**Table 1:** Included games List of included games with year released, player counts, playtime, audience, content area and online links as available. Games with an associated publication either from from the original data source [2] or subsequently identified are cited in the name column.

Year	Name	Players	Playtime	Audience	Content	Webpage
1980	Triage: The Game For Health Care Professionals	2-4	45 min	-	Emergency Medicine	https://boardgamegeek.com/boardgame/136553/triage-the-game-for-health-care-professionals
1992	Friday Night at the ER [20]	4-200	90 min	-	Systems	https://fridaynightattheer.com/
1995	Anatomy Rummy	2-4	-	Student	Anatomy	https://boardgamegeek.com/boardgame/65414/anatomy-rummy
2004	The Last Straw! [13]	4-25	-	Student	Public Health	https://boardgamegeek.com/boardgame/62134/the-last-straw-a-board-game-on-the-social-determin
2006	The “Three D's” of Cognitive Impairment [32]	3-8	30-45 min	Student	Psychiatry	https://www.mededportal.org/doi/10.15766/mep_2374-8265.243
2009	A Game for Teaching Antimicrobial Mechanisms of Action [36]	2-5	40-90 min	Student	Infectious diseases	https://www.tandfonline.com/doi/full/10.1080/01421590802637958
2010	GER-ANIUM [33]	4-6	90 min	Resident Attending	Geriatrics	https://www.mededportal.org/doi/10.15766/mep_2374-8265.7900
2012	Stop The Pressure	2-12	45-60 min	-	Burns & Plastics	https://shop.focusgames.com/collections/harm-free-care/products/stop-the-pressure
2013	Harm Free Game	2-12	45-60 min	-	Systems	https://shop.focusgames.com/collections/harm-free-care/products/harm-free-game
2013	Occam's Razor	1-6	-	Student	General Medicine, USMLE	https://boardgamegeek.com/boardgame/150794/occams-razor-diagnosticians-dilemma
2014	Anatomy & Physiology Trivia	2-12	60-90 min	Student	General Medicine, Anatomy	https://www.thegamecrafter.com/games/anatomy-physiology-trivia
2015	Debriefing Game Cards [39]	-	120 min	Resident Attending	Interpersonal and Communication Skills	https://links.lww.com/SIH/A216
2015	Aftershock [23]	1-12	90-120 min	Student	Disaster Medicine	https://www.thegamecrafter.com/games/aftershock
2015	The Lesion: Charcot's Tournament	2-4	45-90 min	-	Neurology, Anatomy	https://www.thegamecrafter.com/games/the-lesion:-charcot-s-tournament,
2016	Infection Control the Game	6-14	45-60 min	-	Infectious diseases, Systems	https://shop.focusgames.com/collections/infection-prevention/products/infection-control-game
2016	ECG Bingo	4-30		-	Cardiology	https://em3.org.uk/foamed/30/8/2016/ecg-bingo
2016	The Toxicology Game	3+	20-30 min	-	Toxicology	https://em3.org.uk/foamed/4/7/2016/the-toxicology-game
2016	Burn Game	4-12	45-60 min	Student	Burns & Plastics	https://shop.focusgames.com/collections/harm-free-care/products/burns-game
2016	Sepsis	2-12	45-60 min		Infectious diseases	https://shop.focusgames.com/products/the-sepsis-game?variant=7634693095471
2017	Doctor Jargon - Pediatrics	3-8	-	Student Resident Attending	Communication skills, Pediatrics	https://drjargon.com/
2017	The Game of Hospitals [40]	120	90 min	Student	Systems	https://onlinelibrary.wiley.com/doi/full/10.1111/tct.12615#support-information-section
2017	Game of Stools	2-8	30 min	-	Infectious diseases, Systems	https://shop.focusgames.com/collections/infection-prevention/products/game-of-stools
2017	Stroke Game	2-12	45-60 min	-	Neurology	https://shop.focusgames.com/collections/stroke/products/the-stroke-game
2017	Meducational	4	-	Student Resident Attending	General Medicine	https://www.kickstarter.com/projects/1103157999/meducational-a-card-game-for-medics
2018	Drug Round	2-12	45-60 min	Student Resident Attending	Pharmacology, Systems	https://shop.focusgames.com/collections/harm-free-care/products/drug-round-game
2018	Explain It	-	-	-	Communication skills	https://brokentoydotblog.wordpress.com/home/shared-meded-resources/
2018	Which Job Next - EM	-	-	Student Resident Attending	Task prioritization	https://brokentoydotblog.wordpress.com/home/shared-meded-resources/
2018	Doctor Jargon - Genomics	3-8	-	Student Resident Attending	Communication skills, Genetics	https://drjargon.com/
2018	Cards Against Orthopaedics	-	30-45 min	-	Orthopedics	https://em3.org.uk/foamed/15/6/2018/cards-against-orthopaedics
2018	Musculoskeletal Basics [25]	-	-	Resident Attending	Internal medicine, Rheumatology, Orthopedics	https://www.mededportal.org/doi/10.15766/mep_2374-8265.10749
2018	Portocaval Anastomosis Card Game [34]	-	-	Student	Anatomy, Gastroenterology	https://www.mededportal.org/doi/full/10.15766/mep_2374-8265.10696
2018	GridlockED [18]	2-6	60-90 min	Student Resident Attending	Emergency Medicine	https://www.gridlockedgame.com/
2018	Liver Land!	3-17	30-60 min	Student	Gastroenterology	https://www.thegamecrafter.com/games/liver-land-
2019	Games Squared [19]	2-4	-	-	Education	http://bit.ly/GamesSquaredGME
2019	Which Job Next - Ward	-	-	Student Resident Attending	Task prioritization	https://brokentoydotblog.wordpress.com/home/shared-meded-resources/
2019	The Zombie Sepsis Game	4-16	-	Resident Attending	Emergency Medicine, Infectious disease	https://em3.org.uk/foamed/24/5/2019/the-zombie-sepsis-game
2019	Cards Against Paediatric Dermatology	1+	40 min	-	Pediatrics, Dermatology	https://em3.org.uk/foamed/9/8/2019/cards-against-paediatric-dermatology
2019	Drug Calculation Card	1-4	15-20 min	Student Resident Attending	Pharmacology	https://shop.focusgames.com/collections/harm-free-care/products/drug-calculation
2019	MedGunner	1-4	60-90 min	Student	General Medicine, USMLE	https://www.thegamecrafter.com/games/medgunner-board-prep-:-the-card-game
2019	The Floor	1-12	90 min	Student Resident Attending	Systems	https://thefloorgame.com/
2019	Domestic abuse game	2-12	45-60 min	Student Resident Attending	General Medicine	https://www.dvagame.co.uk/
2019	TriagED	2-3	30-60 min	Student Resident Attending	Emergency Medicine	https://www.gridlockedgame.com/triaged-game
2019	Beat the Shock Clock [16]	4-5	-	Resident Attending	Pediatrics, Emergency Medicine	https://www.mededportal.org/doi/10.15766/mep_2374-8265.10804
2019	L&D in the ED [26]	3-4	40 min	Student Resident Attending	Obstetrics, Emergency Medicine	https://www.mededportal.org/doi/10.15766/mep_2374-8265.10815
2020	Cards Against Paediatric ECGs	1+	30-45 min	-	Pediatrics, Cardiology	https://em3.org.uk/foamed/23/9/2020/cards-against-paediatric-ecgs
2020	Cards Against Paediatric Orthopaedics (Upper Limbs)	1+	60-90 min	-	Pediatrics, Orthopedics	https://em3.org.uk/foamed/25/6/2021/cards-against-paediatric-orthopaedics-upper-limbs
2020	The Paediatric Foreign Body Game	-	-	-	Pediatrics, Emergency Medicine	https://em3.org.uk/foamed/6/10/2020/the-paediatric-foreign-body-game
2020	First Aid the Game	2-12	45-60 min	-	General Medicine	https://shop.focusgames.com/collections/harm-free-care/products/first-aid-game
2020	Empiric Emergency Medicine [15]	2-4	15 min	Student Resident Attending	Emergency medicine, Infectious diseases	https://www.drivethrucards.com/product/329118/Empiric-Emergency-Medicine
2020	SPRINT [27]	2-3	30 min	Resident Attending	Emergency Medicine, Task Prioritization	https://www.mededportal.org/doi/10.15766/mep_2374-8265.10956
2020	Clinical Coaching Cards [28]	-	30-90 min	Student Resident Attending	Education	https://www.mededportal.org/doi/10.15766/mep_2374-8265.11042
2020	The Bloody Board Game [29]	28	60-90 min	Resident Attending	Hematology	https://www.mededportal.org/doi/10.15766/mep_2374-8265.11057
2020	Name that Nerve	2-8	30 min	Student	Anatomy	https://www.thegamecrafter.com/games/name-that-nerve
2020	Retain [24]	2-6	30-60 min	-	Neonatology, Pediatrics	https://retainlabsmedical.com
2021	AMS game	4-14	45-60 min	Student	Infectious diseases	https://amsgame.com/
2021	Cards Against Paediatric Orthopaedics (Lower Limbs)	1+	60-90 min	-	Pediatrics, Orthopedics	https://em3.org.uk/foamed/2/7/2021/cards-against-paediatric-orthopaedics-lower-limbs
2021	Tablerounds	2+	-	Student	General Medicine	https://playtablerounds.com/product/table-rounds/
2021	Infection Management	2-8	20-40 min	Resident Attending	Infectious diseases, Systems	https://shop.focusgames.com/collections/infection-prevention/products/infection-management
2021	Empiric Outpatient Medicine [15]	2-4	15 min	Student Resident Attending	Internal Medicine, Infectious diseases	https://www.empiricgame.com/
2022	Candy Gland [30]	6-8	50 min	Student	Endocrinology	https://www.mededportal.org/doi/10.15766/mep_2374-8265.11294
2022	BacteriaGame	3-10	30-60 min	Student Resident Attending	Infectious diseases	https://www.sfm-microbiologie.org/boutique/bacteriagame/
2022	Cranial Vault	2-10	30 min	-	Neurology	https://www.thegamecrafter.com/games/cranial-vault
2022	Foramina!	2-4	30-60 min	-	Neurology, Anatomy	https://www.foraminagame.com/
2022	Gut It Out	2-6	30-60 min	Student	Anatomy	https://www.catlau.ca/gut-it-out
2022	NeuroNavigator	1-4	30-60 min	-	Neurology, Anatomy	https://www.austinlim.com/games
2023	Rooticle	3-5	30 min	-	Neurology, Anatomy	https://www.thegamecrafter.com/games/rooticle
2023	Wordotomy	2-8	20-40 min	Student	General Medicine	http://wordotomy.com/
2023	Pharmageddon: Bugs vs Drugs	1-4	20-30 min	Student Resident Attending	Infectious diseases	https://boardgamegeek.com/boardgame/357797/pharmageddon-bugs-vs-drugs
2023	Medicos	3+	-	Student	General Medicine	https://medicosgame.org/
2023	Empiric Inpatient Pediatrics [15]	2-4	15 min	Student Resident Attending	Pediatrics, Infectious diseases	https://www.drivethrucards.com/product/408190/Empiric-Inpatient-Pediatrics
2023	Empiric Pediatrics 2023 [15]	2-4	15 min	Student Resident Attending	Pediatrics, Infectious diseases	https://www.drivethrucards.com/product/451853/Empiric-Pediatrics-2023
2023	Essential Diagnosis	2	10-15 min	Student Resident Attending	General Medicine	https://www.essentialdiagnosis.com/
2023	Billing Bonanza [17]	2-4	20 min	Resident Attending	Administrative	https://www.mededportal.org/doi/10.15766/mep_2374-8265.11307
2023	TaskMaster [37]	3-24	50-50 min	Student	Task prioritization	https://www.mededportal.org/doi/10.15766/mep_2374-8265.11373
2023	Neurospeed [14]	3-10	40-45 min	Student	Neurology	https://www.neurologygames.com/
2023	Neurologic Hats [21]	3-10	40-45 min	Student	Neurology	https://www.neurologygames.com/
2023	Stroke of Genius [35]	3-5	60-90 min	Student Resident Attending	Neurology	https://boardgamegeek.com/boardgame/378852/stroke-genius
2024	What pharma game	2-6	20-30 min	Student	Pharmacology	https://whatthepharmagame.com/
2024	Bed race [22]	2-4	45 min	Student	Palliative Care	https://www.bedracegame.com/
2024	Livogena [31]	6-150	60 min	Student	Gastroenterology, Hematology	https://www.mededportal.org/doi/10.15766/mep_2374-8265.11381
2024	Patient Communication Challenge [38]	-	40 min	Student	Communication skills	https://www.mededportal.org/doi/10.15766/mep_2374-8265.11411
2024	Antibiogram Slam	3-10	30-60 min	Student Resident Attending	Infectious diseases	https://www.thegamecrafter.com/games/antibiogram-slam-
2024	Field Cut	2-2	30 min	-	Anatomy, Neurology	https://www.thegamecrafter.com/games/field-cut
2024	Organ-ize renal	1+	30 min	Student	Anatomy	https://www.thegamecrafter.com/games/organ-ize
2024	Till death	1-99	30-90 min	-	Pharmacology	https://www.thegamecrafter.com/games/till-death
Unknown	Assessing Arrhythmias	1-4	10 min	-	Cardiology	https://boardgamegeek.com/boardgame/65424/assessing-arrhythmias
Unknown	Virogame	-	-	Student	Infectious diseases	https://www.sfm-microbiologie.org/boutique/virogame/
